# Krumholzibacteriota and Deltaproteobacteria contain rare genetic potential to liberate carbon from monoaromatic compounds in subsurface coal seams

**DOI:** 10.1128/mbio.01735-23

**Published:** 2024-02-12

**Authors:** Bronwyn C. Campbell, Paul Greenfield, Elliott P. Barnhart, Se Gong, David J. Midgley, Ian T. Paulsen, Simon C. George

**Affiliations:** 1 Environment Business Unit, Commonwealth Scientific and Industrial Research Organisation (CSIRO), Floreat, Western Australia, Australia; 2 School of Natural Sciences, Macquarie University, North Ryde, New South Wales, Australia; 3 Energy Business Unit, Commonwealth Scientific and Industrial Research Organisation (CSIRO), Lindfield, New South Wales, Australia; 4 U.S. Geological Survey, Wyoming-Montana Water Science Center, Helena, Montana, USA; University of Tennessee at Knoxville, Knoxville, Tennessee, USA

**Keywords:** coal seam microbiome, dearomatization, biodegradation, benzoyl-CoA reductase, Krumholzibacteriota, coal seam microbiology, coal bed methane, microbial ecology, deep subsurface, aromatic hydrocarbons, carbon cycling, metagenomics

## Abstract

Biogenic methane in subsurface coal seam environments is produced by diverse consortia of microbes. Although this methane is useful for global energy security, it remains unclear which microbes can liberate carbon from the coal. Most of this carbon is relatively resistant to biodegradation, as it is contained within aromatic rings. Thus, to explore for coal-degrading taxa in the subsurface, this study reconstructed relevant metagenome-assembled genomes (MAGs) from coal seams by using a key genomic marker for the anaerobic degradation of monoaromatic compounds as a guide: the benzoyl-CoA reductase gene (*bcrABCD*). Three MAGs were identified with this genetic potential. The first represented a novel taxon from the Krumholzibacteriota phylum, which this study is the first to describe. This Krumholzibacteriota MAG contained a full set of genes for benzoyl-CoA dearomatization, in addition to other genes for anaerobic catabolism of monoaromatics. Analysis of Krumholzibacteriota MAGs from other environments revealed that this genetic potential may be common, and thus, Krumholzibacteriota may be important organisms for the liberation of recalcitrant carbon in a broad range of environments. Moreover, the assembly and characterization of two *Syntrophorhabdus aromaticivorans* MAGs from different continents and a *Syntrophaceae* sp. MAG implicate the Deltaproteobacteria class in coal seam monoaromatic degradation. Each of these taxa are potential rate-limiting organisms for subsurface coal-to-methane biodegradation. Their description here provides some understanding of their function within the coal seam microbiome and will help inform future efforts in coal bed methane stimulation, anoxic bioremediation of organic pollutants, and assessments of anoxic, subsurface carbon cycling and emissions.

**IMPORTANCE:**

Subsurface coal seams are highly anoxic, oligotrophic environments, where the main source of carbon is “locked away” within aromatic rings. Despite these challenges, many coal seams accumulate biogenic methane, implying that the coal seam microbiome is “unlocking” this carbon source *in situ*. For over two decades, researchers have endeavored to understand which organisms perform these processes. This study provides the first descriptions of organisms with this genetic potential from the coal seam environment. Here, we report metagenomic insights into carbon liberation from aromatic molecules and the degradation pathways involved and describe a Krumholzibacteriota, two *Syntrophorhabdus aromaticivorans*, and a *Syntrophaceae* MAG that contain this genetic potential. This is also the first time that the Krumholzibacteriota phylum has been implicated in anaerobic dearomatization of aromatic hydrocarbons. This potential is identified here in numerous MAGs from other terrestrial and marine subsurface habitats, implicating the Krumholzibacteriota in carbon-cycling processes across a broad range of environments.

## INTRODUCTION

The global energy transition from hydrocarbons to renewables requires low-emission fuels for facilitating energy security during this shift. For this transition fuel need, methane is one such lower emission alternative to coal (1). Methane gas provides a dispatchable source of energy that, unlike coal, produces neither particulates nor harmful nitrous and sulfur oxides during combustion (2, 3). Thus, significant research interest exists in enhancing rates of methane gas production in subsurface coal by using the coal seam microbiome (4–6).

Within the coal itself, carbon is primarily (~60% to 100%) contained within aromatic rings, which increase in abundance with thermal maturity (7). Thus, microorganisms capable of aromatic ring degradation may be the dominant contributors of carbon to the coal seam microbiome, especially in more thermally mature coals. Over the last decade, an understanding has been formed of the range of microbes that occur in subsurface coal seams, demonstrating that they are typically characterized by a few relatively abundant taxa and a long-tailed distribution of rarer taxa (4, 8, 9). Coal seam microbiome studies have also revealed that the dominant organisms, such as taxa within *Desulfuromonas* and *Desulfovibrio*, are not involved in aromatic degradation but rather degrade simpler intermediates (8, 10). Aromatic-degrading taxa, therefore probably occur within the rarer taxa.

Identification of aromatic-degrading taxa is important both for enhancing applied outcomes such as industrial gas production and for understanding carbon mobilization from carbon-rich regions of the lithosphere. Indeed, the research effort to identify these taxa has been ongoing in the subsurface coal microbiology field for the last three decades (4, 5, 8, 11). Some taxa have previously been identified with the genetic tools for aerobic aromatic ring degradation within coal seams (12–14); however, aerobic pathway contributions are likely restricted to very shallow regions of meteoric water infiltration, since subsurface coal seams are overall highly anoxic environments (14). Within other anoxic hydrocarbon-degrading environments, such as oil reservoirs (15), the catabolism of aromatic substrates progresses at least in part via intermediate aromatic compounds. Although a wide range of monoaromatic biodegradation pathways are known, these pathways are dependent upon a relatively small range of central intermediate aromatic compounds. One intermediate aromatic compound in particular, known as benzoyl coenzyme A (benzoyl-CoA), is central to the anaerobic degradation of a particularly wide range of monoaromatics (16). Further catabolism from this central intermediate requires the benzoyl-CoA reductase enzyme, which is responsible for the first dearomatization steps of the ring (17). This enzyme for ring cleavage is a crucial step for accessing the carbon contained within the ring structures, as the thermodynamic stability of the aromatic ring structure renders it highly resistant to degradation (15). The ability to encode this enzyme, and those for the proceeding metabolites in the benzoyl-CoA pathway, would provide organisms with access to much of the carbon locked up in the organic substrates present in coal.

To date, numerous studies have attempted to identify aromatic-degrading taxa in coal seams using a range of strategies. Some of these have included the enrichment of microbes capable of degrading aromatic compounds, using either a variety of mono- and polyaromatic compounds or organic matter of differing maturities as sole sources of carbon (18, 19). These studies identified putative aromatic degraders among the enriched taxa but also obscured primary aromatic degraders amid the numerous taxa subsequently enriched on their downstream degradative products.

Metagenome-assembled genomes (MAGs) have also been used to identify putative aromatic-degrading taxa by using known genes required for hydrocarbon degradation, performed alongside methods such as bio-orthogonal non-canonical amino acid tagging to differentiate active from inactive cells (12, 13). Within the active taxa, this strategy has identified a range of genes used for rearranging the substituents of the aromatic ring in peripheral pathways above the benzoyl-CoA intermediate, for example, genes for catabolism of ethylbenzene to acetophenone in *Chlorobiota* and *Geobacter* taxa (12). Although these studies identify an abundance of genes and taxa actively involved in the coal-to-methane degradation pathways, the specific anaerobic monoaromatic-degrading genes identified are not involved in catalyzing dearomatization reactions or capable of liberating carbon from their targeted substrates (15, 20, 21). Consequently, the identification of taxa containing the genes for dearomatization of these monoaromatic substrates, such as the benzoyl-CoA reductase gene, remains an unanswered and critical step for understanding the *in situ* liberation of carbon from coal.

Recently, *Syntrophorhabdaceae* and *Syntrophaceae* were implicated as potentially important coal-degrading families using the linear discriminant analysis effect size statistical method on a group of algal-amended coal seam microbiomes from the Powder River Basin, USA (22). *Syntrophorhabdaceae* is a monotypic family, of which *Syntrophorhabdus aromaticivorans* is presently the sole described species (23). *S. aromaticivorans* has previously been identified within an enriched Surat Basin (Australia) coal seam microbial community, which had likely responded to the increased surface area of the provided coal that had undergone solvent extraction (24). Outside the coal seam environment, an *S. aromaticivorans* isolated from an anaerobic digester was demonstrated to anaerobically catabolize multiple monoaromatic substrates to acetate, to utilize a model organic electron acceptor, and was proposed to be capable of interspecies electron transfer in partnership with a hydrogenotrophic methanogen (23). *S. aromaticivorans* is thus a promising candidate for monoaromatic degradation capabilities within the coal seam environment. In contrast to this, *Syntrophaceae* spp. are more commonly associated with aliphatic degradation than aromatic degradation; however, some taxa within the family can utilize a limited range of more labile monoaromatic substrates (25, 26). Although *Syntrophaceae* spp. and *S. aromaticivorans* have been implicated as taxa that may be important for *in situ* coal degradation, there has been no demonstration that strains from this environment have the genetic tools required to access aromatic compounds or culture-based demonstrations of their activity against aromatic constituents of coal.

One alternative strategy to those outlined above is to “mine” metagenomic data for specific aromatic-degrading genes and then use binning techniques to reassemble genomes of potential aromatic degraders. Accordingly, this study aimed to identify putative aromatic-degrading taxa by mining metagenomic data from Australia and North America through first identifying assembled DNA sequences that contained benzoyl-CoA reductase gene subunits and then using these as references to assemble MAGs for the identification of these taxa and their potential capabilities.

## MATERIALS AND METHODS

### Sources of metagenomic DNA

For further details of metagenomic sequence data sourcing, see reference 27. Briefly, whole-genome shotgun sequences selected for use were required to (i) be from a subsurface coal seam (ii), state the coal seam or associated geological basin, (iii) have been sequenced using Illumina (28), and (iv) be available as unassembled sequence reads. Nine metagenomes from North America and four from Australia were found to be suitable (Table S1). Eight of the North American metagenomes were from the Nance, Flowers-Goodale, and Terret coal seams of the Powder River Basin, USA, and one was from the Pocahontas No. 3 Coal of the central Appalachian Basin, USA. From Australia, three metagenomes were from the Walloon Subgroup of the Surat Basin, and one was from the Bandanna Formation of the underlying Bowen Basin, and all of these from the state of Queensland, eastern Australia.

### Reassembly, annotation, and detection of contigs containing benzoyl-CoA reductase gene subunits

All metagenomes were downloaded as unassembled reads and passed through the same error correction, assembly, and annotation pipeline. These reads were error corrected using Blue v2 (29) prior to assembly using SPAdes v3.13.2 with the meta flag (30). Resultant contigs were annotated using prokkaMeta v1.14.5 (31), and these annotation descriptions were then searched for any class-I enzyme system benzoyl-CoA reductase subunits, which are associated with facultative anaerobes such as *Magnetospirillum* and *Thauera* spp. (15, 32). Contigs with benzoyl-CoA reductase gene subunits were then also explored, using the Prokka annotations, for other genes in the benzoyl-CoA pathway. The contigs on the final list selected for further analysis all contained at least one benzoyl-CoA reductase gene subunit (*bcrABCD*), were of lengths greater than 7 kbp, and had coverages greater than five.

### Generation of trimer signatures, correlations, bin quality control, and accessioning

In order to identify other contigs within the metagenome from the same taxon, a trimer approach was used (33). In brief, the Python programming language (34) was used to count the proportion of each of the 64 possible trimers in all contigs containing at least 1,000 base pairs. The trimer signature for those contigs containing benzoyl-CoA reductase gene subunits was then used as a reference, and the trimer signatures for all contigs in the metagenome were examined against these reference contigs using a Pearson correlation coefficient in SciPy 1.7.3 (35). Those contigs with Pearson’s *R* values greater than 0.95 were collected into bins for quality control. As a subsequent step in bin quality control, the coverages of each contig within the bin were inspected manually, and contigs with aberrant coverage were removed. Each bin was then subject to inspection for completeness and contamination using CheckM v1.1.3 (36).

### Characterization of the genomic content of the bins

Summary contig statistics (total bin length, total contigs, mean contig length, median contig length, N50, maximum contig length, and GC content) were determined using Python. The bins were then submitted to online tools to further characterize the genomic content of each bin. For use with KEGG Mapper (genome.jp/kegg/mapper) (37), the Prokka-annotated amino acid files were submitted to the BlastKOALA v2.2 online tool and run against the “species_prokaryotes” database (38). The Prokka-annotated amino acid files were also used to determine which transport proteins were present in each bin, by submission to the TransportDB 2.0 TransAAP (membranetransport.org) for transporter annotation (39). In order to characterize the carbohydrate active enzymes, dbCAN HMMdb v10.0 (bcb.unl.edu/dbCAN2) was run on the unannotated contigs within each bin (40). Similarly, the CRISPR sequences and *cas* genes were identified in the contigs within each bin using CRISPRCasFinder online (crisprcas.i2bc.paris-saclay.fr) (41). Resultant data from these tools were summarized and used to characterize the genomes in each bin and estimate their ecological roles within the coal seam environment.

### Identification of the bins

As all bins lacked 16S ribosomal RNA (rRNA) genes, the elongation factor G genes were used to identify the closest known relatives of each bin. For this, BLASTx searches of the non-redundant protein database were used (https://blast.ncbi.nlm.nih.gov/Blast.cgi). Subsequently, putative identities from elongation factor G genes were compared with BLASTN searches of 16S rRNA (V4) genes obtained from each metagenome using the Earth Microbiome Project V4 region primers with Kelpie (42–44). This manual comparison between the elongation factor G taxonomy and Kelpie-derived 16S rRNA genes was used to identify putative taxa at the level of family or lower, with a minimum per-taxon abundance of 10 applied for the 16S rRNA genes. In addition, all 16S rRNA genes generated using Kelpie were then compared to the coal seam microbiome (CSMB) reference set, using USEARCH v11.0.667 at 97% identity, to determine any associated CSMB reference taxa (8, 45).

Where close elongation factor G sequence percent identities were not found with BLASTx, the phylogeny of the bin was estimated against representative genomes using multilocus sequence analysis (MLSA). Relevant representative genome assemblies and MAGs were sourced from the NCBI Assembly database (ncbi.nlm.nih.gov/assembly/) (46) and annotated using Prokka. Common housekeeping genes were then compiled using a Python script (34), with their selection restricted to those housekeeping genes present within both the bin and the majority of the representative genomes. The constructed fasta files of the selected housekeeping genes were aligned and clustered in Mega v11.0.13 (47) with the ClustalW function and edited as Newick tree format files in FigTree (tree.bio.ed.ac.uk/software/figtree/).

### Figures

Final editing of each figure was performed using Inkscape v1.1.2 (48) or Adobe Illustrator v26.0.2 (49).

## RESULTS

### Distribution of benzoyl-CoA reductase genes across the examined metagenomes

Subunits of the benzoyl-CoA reductase gene (*bcrABCD*) were detected in the Prokka annotations of all examined metagenomes except for the Appalachian Basin metagenome (Table S2a). Relative to the total coding sequences (CDS) identified in each metagenome, the proportion of *bcrABCD* subunits per million CDS ranged from 17 to 52, with Powder River 85 (Flowers-Goodale coal seam) containing the lowest proportion and Powder River 10 (Nance coal seam) containing the highest relative proportion. This is not a measure of relative abundance of these genes but rather an indication of the number of times the *bcrABCD* subunits were identified within larger distinct fragments of assembled contiguous sequences (contigs). Overall, subunits *bcrB* and *bcrC* were identified far more often than subunits *bcrA* and *bcrD*; no *bcrA* or *bcrD* subunits were detected in the Australian metagenomes, and the proportion of *bcrBC* subunits was an order of magnitude higher than *bcrAD* in the Powder River Basin metagenomes (218 and 27 per million CDS, respectively).

**Fig 1:**
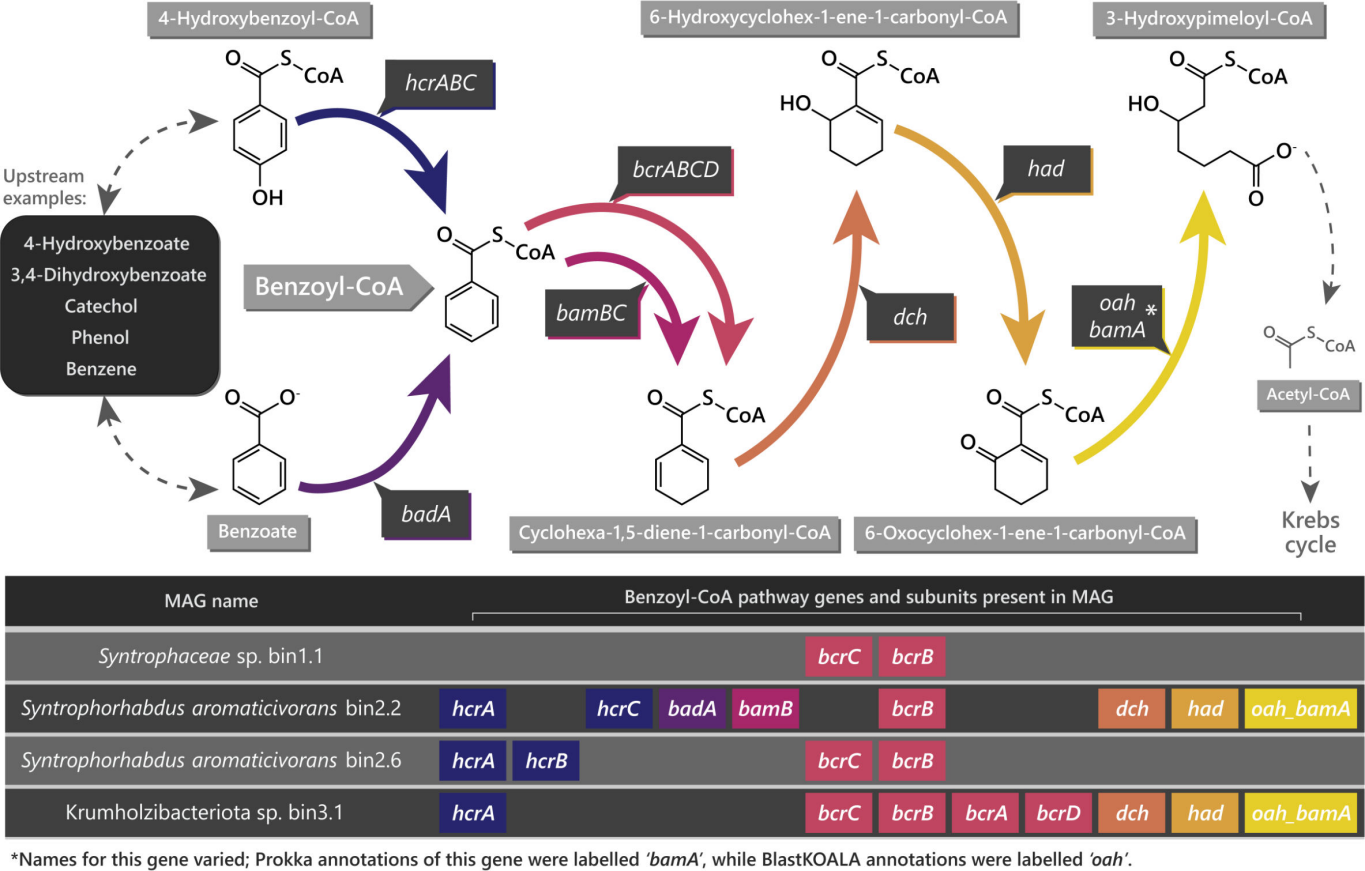
Benzoyl-CoA pathway genes and subunits, from 4-hydroxybenzoyl-CoA and benzoate to 3-hydroxypimeloyl-CoA, and their presence in the selected contig bins (MAGs). Benzoyl-CoA reductase genes for two enzyme systems are displayed: the class-I, ATP-dependent *bcrABCD* (associated with facultative anaerobes), as well as the class-II, ATP-independent *bamB* (associated with obligate anaerobes) (15, 32). See Table S2 for number of occurrences of each gene in the MAGs and metagenomes. The displayed benzoyl-CoA pathway was adapted from genome.jp/pathway/map00362 (37).

Twenty-one bins were obtained that contained benzoyl-CoA reductase gene subunits, and pair-wise comparison of these indicated three groupings of similar genomes (Table S3). A representative bin was chosen from each of these groupings, with the highest completeness and lowest contamination scores in the CheckM results for that group. Although groups 1 and 3 contained bins only from the Powder River Basin, group 2 contained bins from the Powder River, Bowen, and Surat basins, so a representative bin from each continent was selected for further characterization (bins 1.1, 2.2, 2.6, and 3.1; Fig. 1; Table 1). The remaining 17 bins were determined to be chimeric, as they were too large to represent a single genome or to have unacceptably low completeness and/or unacceptably high contamination scores in the CheckM results (Table S3).

**TABLE 1:** Bin statistics

Parameter	*Syntrophaceae* sp. bin 1.1	*S. aromaticivorans* bin 2.2	*S. aromaticivorans* bin 2.6	Krumholzibacteriota bin 3.1
Summary statistics category				
Total length (Mbp)	2.15	2.19	1.96	4.18
Total contigs	10	36	19	45
Mean contig length (Mbp)	0.22	0.06	0.1	0.09
Median contig length (Mbp)	0.16	0.05	0.08	0.07
N50	0.39	0.08	0.11	0.14
Maximum contig length (Mbp)	0.5	0.15	0.25	0.56
GC content (%)	47.6	44.6	47.9	71.1
CheckM results (%) (36)				
Completeness	59.35	77.42	54.62	94.51
Contamination	0.65	1.29	1.68	1.1

### Phylogeny of the four contig bins that were chosen for further analysis

16S rRNA genes were not recovered in any of the four contig bins selected for further analysis or in the other related 17 bins. In place of this, phylogenetic analyses were performed using BLASTx to identify elongation factor G gene sequences with high percent identities to the four contig bins and then infer the associated 16S rRNA gene operational taxonomic units (OTUs). These results indicated that the genomic content of these four bins came from a *Syntrophaceae* species (bin 1.1), two *Syntrophorhabdus aromaticivorans* (bins 2.2 and 2.6), and a novel member of the Fibrobacterota-Chlorobiota-Bacteroidota (FCB) superphylum (bin 3.1). Although the closest relative of bin 1.1 by its elongation factor G gene was a *Syntrophaceae* sp., possible *Syntrophaceae* matches for this bin in the 16S rRNA gene results from the metagenome were limited to two distinct taxa (OTU_64 and OTU_114; detected 12 and 10 times, respectively). Both of these have >90% identities to type sequence *Smithella propionica* LYP (96.84% and 94.86%, respectively; GenBank accession NR_024989.1) as well as to each of the three formally described species within the *Syntrophus* genus (93.68%–95.65% and 92.89%–94.09%, respectively). Accordingly, bin 1.1 was referred to here as a *Syntrophaceae* sp., being closely related to the *Smithella* and *Syntrophus* genera. Under recent alternative phylogenomic classifications of the Deltaproteobacteria, *S. aromaticivorans* and the *Syntrophaceae* sp. fall within a phylum named either Thermodesulfobacteriota or Desulfobacterota, and the lowest common rank of close relatives to bin 1.1 is the Syntrophales order (42, 43). The nomenclature used in the present study does not reflect these proposed updates and instead aligns with current entries for Deltaproteobacteria and *Syntrophaceae* in the LPSN (https://lpsn.dsmz.de/).

Although 16S rRNA sequences for the Deltaproteobacteria MAGs were able to be found by correlating close relatives between the Kelpie-produced 16S rRNA gene results and the annotated elongation factor G genes (*Syntrophaceae* sp. bin 1.1 and *Syntrophorhabdus aromaticivorans* bins 2.2 and 2.6), the novelty of bin 3.1 impeded identification through close relatives.

**Fig 2:**
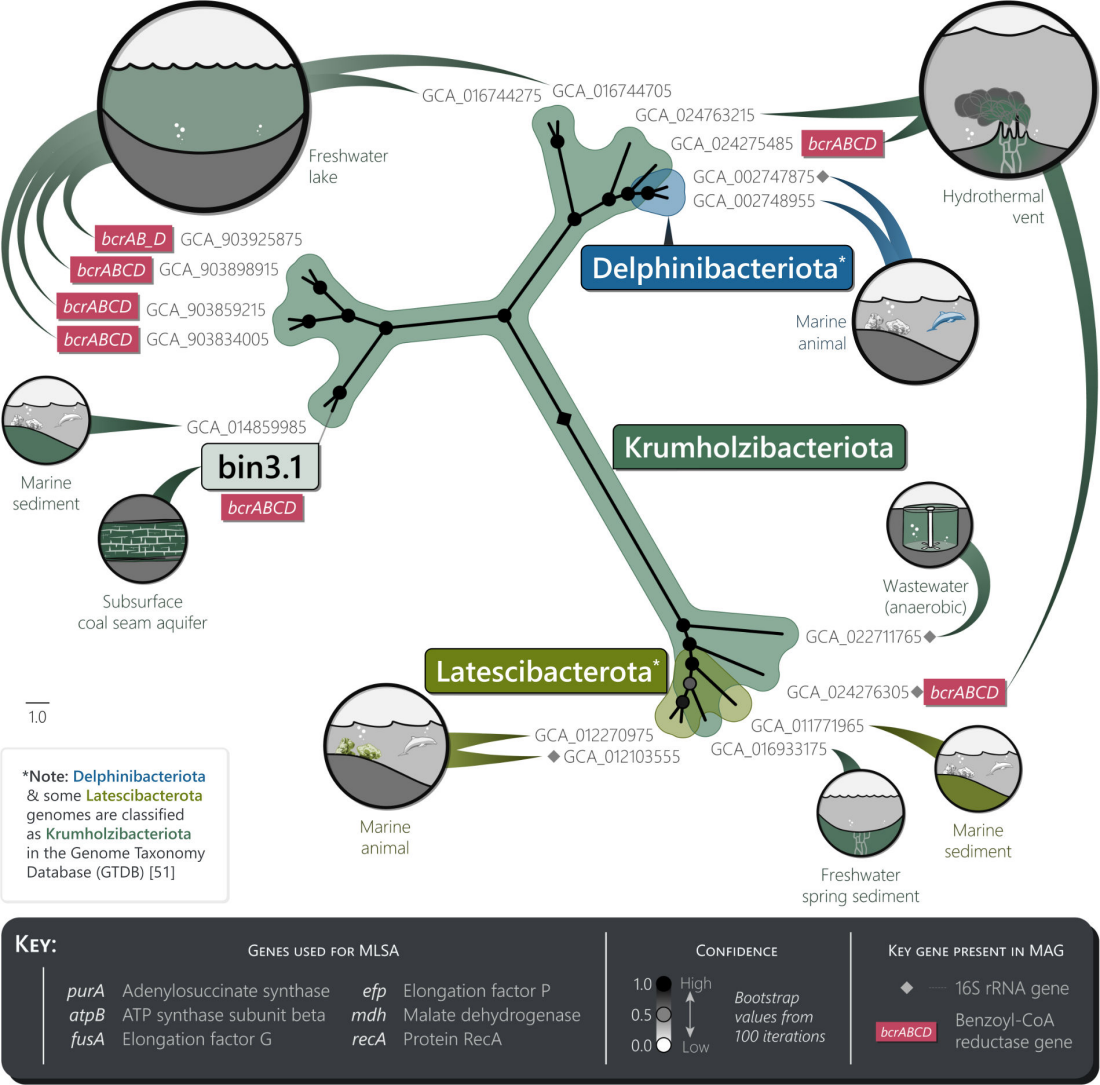
Phylogenetic relationships and environmental sources of selected Krumholzibacteriota, Delphinibacteriota*, and Latescibacterota* MAGs. Maximum likelihood phylogeny of bin 3.1 and 17 representative MAGs using multilocus sequence analysis (MLSA; see Fig. S2 for the equivalent FCB superphylum tree). Thirty other MAGs from the initial analysis were missing the selected housekeeping genes and thus are not included here (Table S4) (52, 54–59). Environmental sources are indicated for each MAG; circle size is proportional to the number of times each environment applied within the same phylum.

MLSA was performed to further characterize bin 3.1 after it was placed loosely within the FCB superphylum by low percent identity NCBI BLAST results using its elongation factor G gene. MLSA was performed first on 37 representative genomes and MAGs from the FCB superphylum (50) and then was again performed on 47 MAGs from the Krumholzibacteriota, Delphinibacteriota, and Latescibacterota candidate phyla once this specific region of the FCB superphylum had been identified as most closely related to bin 3.1 (Fig. S2; Table S4). These phyla are not yet well resolved, and the Genome Taxonomy Database classifies the Delphinibacteriota and some Latescibacterota within the Krumholzibacteriota phylum (51). From these MAGs, six housekeeping genes were selected for analysis: adenylosuccinate synthase, ATP synthase subunit beta, elongation factor G, elongation factor P, malate dehydrogenase, and protein RecA genes, which were identified in bin 3.1 in addition to a maximum number of the representative genomes. Importantly, each of these genes occurred within two or more genomes from Delphinibacteriota and Krumholzibacteriota; the two phyla with which bin 3.1 had the highest percent identities in the BLASTx results for the elongation factor G gene. The results of the MLSA indicate that bin 3.1 is likely a novel member of the Krumholzibacteriota phylum, of which the closest known relative appears to be a marine sediment enrichment culture MAG from the Bothnian Sea, in the Scandinavian region (52). Four other closely related Krumholzibacteriota MAGs were also identified, coming from a stratified freshwater reservoir of the Cañas River in Puerto Rico and from Lake Lovojärvi in Finland (Fig. 2; Table S4b; NCBI Assemblies GCA_ 903834005, GCA_903859215, GCA_903898915, and GCA_903925875, all from project number PRJEB38681) (53).

For the purpose of aiding cross-study analysis of Krumholzibacteriota sp. bin 3.1, the four MAGs in the MLSA results that contained 16S rRNA genes (Fig. 2) were used to determine both the most probable 16S rRNA gene OTU in the present study and the most probable CSMB reference set sequence (8). These four MAGs consisted of a Delphinibacteriota, two Krumholzibacteriota, and a Latescibacterota MAG (NCBI Assemblies GCA_002747875, GCA_022711765, GCA_024276305, and GCA_012103555, respectively). Each of these were compared against the metagenomic 16S rRNA gene OTUs and the CSMB reference set using USEARCH (90% minimum threshold) (45). Although no 16S rRNA gene OTU or CSMB identities were found for Krumholzibacteriota GCA_024276305 or Latescibacterota GCA_012103555, the USEARCH results indicated that Krumholzibacteriota sp. bin 3.1 may correspond to OTU_57 (94.1% and 92.9% identities to GCA_022711765 and GCA_002747875), which also is in agreement with the distribution of similar bin three group MAGs across the metagenomes in the present study (Table S3; assuming the absence of 16S rRNA genes from rarer taxa is due to the shallower sequencing depth overall in Powder River 9). The CSMB identities indicated that Krumholzibacteriota sp. bin 3.1 may correspond to CSMB_1092 (95.5% and 93.9% identities to GCA_022711765 and GCA_002747875), which has been previously reported from coal seams in the Bowen, Surat, and Sydney basins, Australia (Table S5).

### General description of the *Syntrophaceae* sp., *S*. *aromaticivorans*, and Krumholzibacteriota sp. genome bins

The four bins chosen for further characterization ranged in size from 1.96 to 4.18 Mbp, with *S. aromaticivorans* bin 2.6 having the smallest bin size and the Krumholzibacteriota sp. having the largest bin size (Table 1). GC content was broadly similar for the three Deltaproteobacteria MAGs (bins 1.1, 2.2, and 2.6), with an average of 46.7%. Krumholzibacteriota sp. bin 3.1, however, had a higher GC content of 71.1% (Table 1) consistent with the other closely related Krumholzibacteriota MAGs from freshwater lakes and marine sediment (69.2%–70.8%; Fig. 2; Table S4) (52, 53). Across the four bins, CheckM (36) results indicated that contamination was very low (<2%). Although only taxonomically resolved to the phylum level, genome completeness was greatest for Krumholzibacteriota sp. bin 3.1 (95%), whereas genome completeness was lowest for the Powder River Basin-sourced *S. aromaticivorans* bin 2.6 (55%; Table 1; Table S3). Overall, these bins are medium- to high-quality draft MAGs, based on the combination of completeness and contamination scores with the varying presence of key marker genes within the assemblies (60). Although completeness and contamination scores for the Krumholzibacteriota sp. bin 3.1 would support its classification as a high-quality draft, it is instead classified as a medium-quality draft due to the lack of recovered 16S rRNA genes.

### Dearomatization genes and other notable ecophysiological MAG characteristics

The most commonly detected benzoyl-CoA reductase gene subunits were those of *bcrABCD* from the class-I enzyme system, associated with facultative anaerobes such as *Magnetospirillum* and *Thauera* spp. (15, 32). The Prokka (31) annotations contained the highest number of these; however, the BlastKOALA (38) annotations included an additional enzyme system (Table 2; Table S2). This additional enzyme system, found in *S. aromaticivorans* bin 2.2, was the ATP-independent class-II benzoyl-CoA reductase gene subunit, *bamB*, associated with obligate anaerobes (Fig. 1) (32).

**TABLE 2:** The abundance of genes or genetic elements identified in the MAGs

Genetic element [annotation tool] (reference)	*Syntrophaceae* sp. bin 1.1	*S. aromaticivorans* bin 2.2	*S. aromaticivorans* bin 2.6	Krumholzibacteriota sp. bin 3.1
Total *bcrABCD* subunits [Prokka] (31)	2	1	3	4
Total *bcrABCD* subunits [BlastKOALA] (38)	0	0	0	4
Annotation summary [Prokka] (31)				
CDS/ORFs	2002	2121	1908	3342
rRNAs	0	0	0	0
tRNAs	33	27	23	52
tmRNAs	0	1	0	1
Carbohydrate-active enzyme class [dbCAN2] (40)				
Glycoside hydrolases	6	15	6	45
Glycosyltransferases	50	29	25	72
Polysaccharide lyases	0	0	0	0
Carbohydrate esterases	1	5	3	10
Auxiliary activities	6	7	7	2
Carbohydrate-binding modules	5	5	3	14
CRISPR sequences [CRISPRCasFinder] (41)				
CRISPR loci	4	3	0	2
Number of spacers	18	30	0	7
Combined total length	136	104	0	51
*cas* genes total	7	9	0	0
Membrane transporter family [TransportDB] (39)				
ATP-binding cassette transporters	66	108	101	81
F-type ATPase	13	16	22	16
Tripartite ATP-independent periplasmic transporters	0	32	36	1
Solute/sodium symporters	8	13	13	24
Major facilitator superfamily	6	15	12	13
Resistance-nodulation-division family transporters	9	3	10	20
Other	69	82	60	111

In addition to their use for MLSA, the Prokka annotations of the representative MAGs from the FCB superphylum, including those from the Krumholzibacteriota, Latescibacterota, and Delphinibacteriota phyla, were searched for benzoyl-CoA pathway genes. No genes of either the *bcr* or *bam* enzyme systems were detected in the 37 representative FCB superphylum genome assemblies and MAGs from the NCBI Assembly database (Table S4a) (46). Other genes in the benzoyl-CoA pathway were also rare, with the highest number (two) being found in the MAG of *Longimicrobium terrae* CECT 8660, from the Gemmatimonadota phylum, for the production of benzoyl-CoA from 4-hydroxybenzoyl-CoA and benzoate (*hcrABC* and *badA*, respectively). In contrast, the *bcr* gene and other benzoyl-CoA reductase genes were far more common among the 40 representative Krumholzibacteriota MAGs from GenBank that were used for the second MLSA during phylogenetic classification of this MAG. From these representative Krumholzibacteriota MAGs, 16 contained one or more subunits from *bcrABCD*, 12 contained all four subunits, and 21 contained one or more genes or subunits from the benzoyl-CoA pathway as depicted in Fig. 1 (Table S4b; Fig. 2) (52, 54, 55). Indeed, 11 of these Krumholzibacteriota MAGs contained complete or near-complete (missing only one gene) pathways from benzoyl-CoA to 3-hydroxypimeloyl-CoA, as was identified in the Krumholzibacteriota MAG (bin 3.1) from the present study. These 11 representative Krumholzibacteriota MAGs that contained the genes for the benzoyl-CoA pathway are recorded as being sourced from a hydrothermal vent chimney along a mid-ocean ridge in the southwest Indian Ocean and from freshwater lakes in Puerto Rico, Switzerland, and Finland (hydrothermal vents [61]: GCA_024275485, GCA_024276305, and GCA_024277025; freshwater lakes [53]: GCA_903834005, GCA_903845975, GCA_903847545, GCA_903849715, GCA_903850775, GCA_903851775, GCA_903859215, GCA_903898915, GCA_903912525, GCA_903916975, and GCA_903925875).

Aside from the benzoyl-CoA pathway genes, the most notable BlastKOALA results regarded flagellar assembly and chemotaxis, anabolic antimicrobial genes, and nutrient and electron acceptor scavenging (Table S2c). Substantial numbers of genes for flagellar assembly and chemotaxis were detected within Krumholzibacteriota sp. bin 3.1 as well as genes for biosynthesis of antimicrobial substances. Genes for nutrient and electron acceptor scavenging of amino acids, nitrogen compounds, and sulfur compounds were most commonly detected within the *S. aromaticivorans* bins 2.2 and 2.6. The substrates for these nitrogen and sulfur scavenging genes included elemental sulfur, sulfite, trithionate, thiosulfate, ammonia, and nitrogen gas. Krumholzibacteriota sp. bin 3.1 also contained genes for removing amine and phosphate functional groups from monoaromatic compounds.

### Carbohydrate-active enzymes

Each of the four bins contained a small number of carbohydrate-active enzymes (Table 2). The highest number of carbohydrate-active enzymes was detected in Krumholzibacteriota sp. bin 3.1 (143 total), of which the majority were glycosyltransferases and glycoside hydrolases. Of the glycoside hydrolase genes in Krumholzibacteriota bin 3.1, those for starch/glycogen catabolism were most abundant, although genes for oligosaccharides, fructan, cellulose, and other plant and animal polysaccharides were also detected (62). The lowest number of carbohydrate-active enzymes was found in *S. aromaticivorans* bin 2.6 (44 total), more than half of which were glycosyltransferases. Overall, glycosyltransferases were the most commonly detected carbohydrate-active enzymes in each bin. No polysaccharide lyases were detected in any of the bins.

### Membrane transporter proteins

Numerous transporter protein genes were detected in each bin (Table 2; Table S2d). *Syntrophorhabdus aromaticivorans* bins 2.2 and 2.6, along with Krumholzibacteriota sp. bin 3.1, contained higher numbers of transporter proteins (totaling 269, 254, and 266, respectively). In contrast, *Syntrophaceae* sp. bin 1.1 contained a lower number of transporter proteins (totaling 171). Across all four bins, the ATP-binding cassette transporters were the most commonly detected; however, it is also notable that the tripartite ATP-independent periplasmic transporters were unusually abundant within the *S. aromaticivorans* bins 2.2 and 2.6 (32 and 36, respectively). Efflux transporter genes were also found in all bins, although were twice as abundant in Krumholzibacteriota sp. bin 3.1, including three associated with cobalt-zinc-cadmium resistance.

### CRISPRs and *cas* genes

Relevant to viral predation and defense, three of the four bins included multiple CRISPR loci, and two of these (bins 1.1 and 2.2) contained several *cas* genes (Table 2; Table S2e). *Syntrophaceae* sp. bin 1.1 had four CRISPR loci containing 18 spacers, *S. aromaticivorans* bin 2.2 had three CRISPR loci and 30 spacers, and Krumholzibacteriota sp. bin 3.1 had two CRISPR loci and seven spacers. Notably, while *Syntrophaceae* sp. bin 1.1 and *S. aromaticivorans* bin 2.2 contained the aforementioned *cas* genes (7 and 9, respectively), no *cas* genes were detected within Krumholzibacteriota sp. bin 3.1, and neither CRISPR loci nor *cas* genes were detected in *S. aromaticivorans* bin 2.6.

## DISCUSSION

For microbiologists working to understand the catabolism of coal in the subsurface, the identification of taxa engaged in degrading aromatic compounds has proved elusive. Indeed, microbial communities from the coal seam environment tend to be numerically dominated by one or two methanogens (12, 63), a handful of bacterial taxa presumably engaging in syntrophic partnerships with these methanogens, and a long-tailed distribution of rarer taxa (64). Since it has generally been the view that these bacterial syntrophs cannot degrade complex aromatic or aliphatic compounds (4, 65, 66), this leaves the relatively unexplored long tail of the coal seam microbiome as a likely place to find those microbes capable of coal degradation. This unexplored long tail is comprised of low-abundance, diverse taxa, collectively known as the “rare biosphere” (67–69). It is ecologically reasonable to hypothesize that the unexplored rare biosphere of coal seam microbiome contains microbes with a more diverse array of nutritional strategies, since presumably, there are abundant and less-highly competed nutritional niches within the diverse heterogeneity of the organic matter in coal. Data presented here demonstrate for the first time the identity of multiple lineages with crucial monoaromatic-degrading genes from subsurface coal seams on two continents. Four MAGs were explored in this study, identified as a *Syntrophaceae* sp. (bin 1.1), two *Syntrophorhabdus aromaticivorans* (bins 2.2 and 2.6), and a novel taxon from the Krumholzibacteriota phylum in the FCB superphylum (bin 3.1). The Krumholzibacteriota sp. represented by this MAG is likely engaged in monoaromatic degradation within the Flowers-Goodale coal seam of the Powder River Basin and had not previously been identified in the coal seam environment, nor had it been implicated as having a role in carbon liberation from aromatic compounds present in coal. While it has been previously stated that *S. aromaticivorans* may be important for coal degradation (22), this is the first demonstration that coal seam MAGs from this taxon carry genes for monoaromatic degradation. For the *Syntrophaceae* sp., taxa within this family are well-known hydrocarbon degraders in subsurface environments, such as aliphatic molecule degradation in oil reservoirs (26); however, they had not previously been implicated in aromatic degradation in the coal seam environment.

### Ecological characteristics of the Krumholzibacteriota sp. bin 3.1 MAG

**Fig 3:**
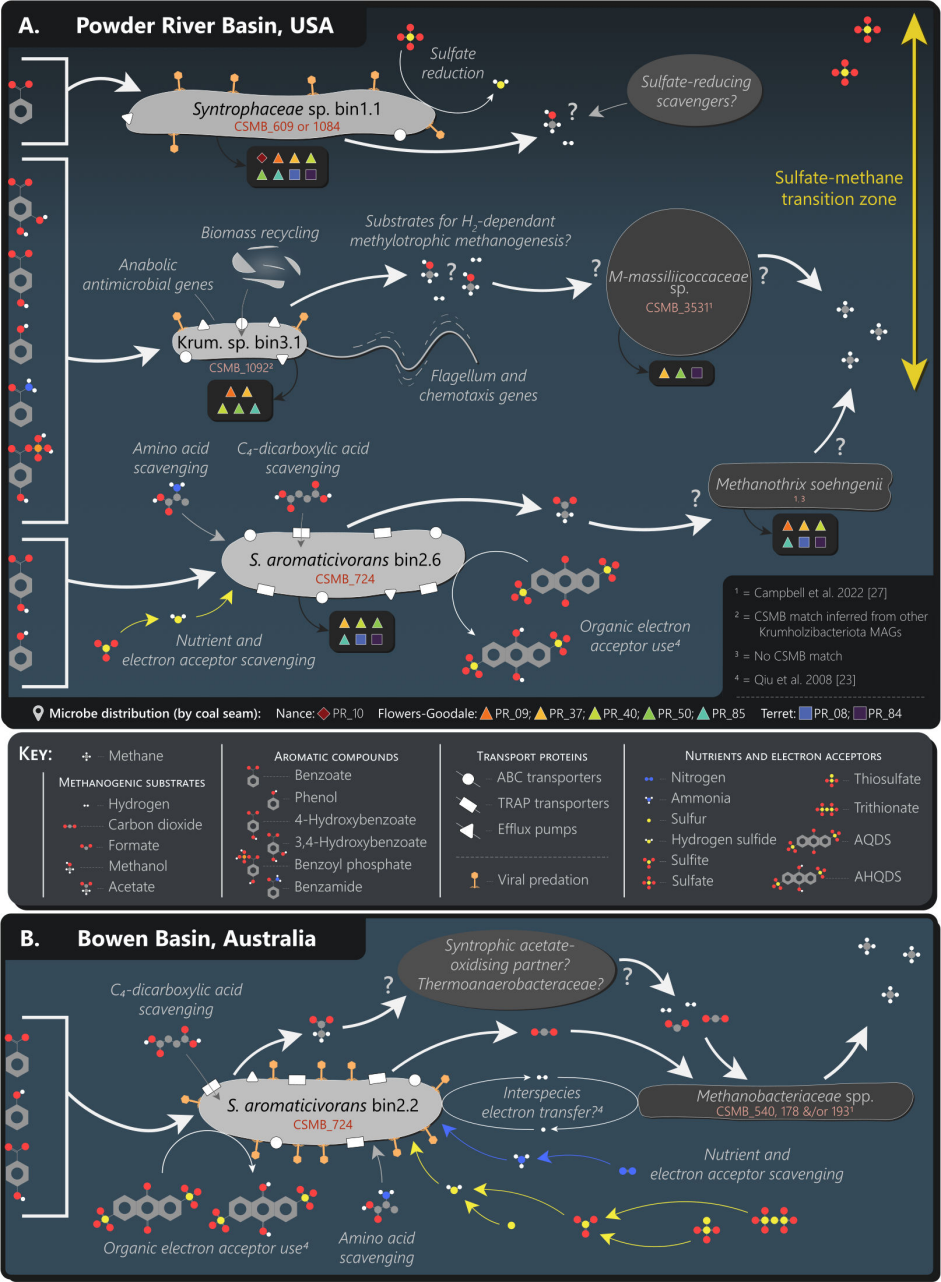
Putative ecological functions within the coal seam microbiome (Table S2). Conceptual model displaying (**A**) the *Syntrophaceae* sp., Krumholzibacteriota sp., and *Syntrophorhabdus aromaticivorans* within the Nance, Flowers-Goodale, and Terret coal seams of the Powder River Basin, USA, and (**B**) the *Syntrophorhabdus aromaticivorans* within the Bandanna Formation of the Bowen Basin, Australia. The most abundant methanogen/s (according to *mcrA* and 16S rRNA gene data) (27) in the metagenome of each bin has been included in the model; however, these are not necessarily the key partner methanogens for the aromatic-degrading taxa.

Based on its genetic potential, the Krumholzibacteriota sp. bin 3.1 MAG represents a primary coal degrader with the ability to anaerobically catabolize a wide range of monoaromatic compounds (Fig. 3; Table S2). This implicates the Krumholzibacteriota candidate phylum in aromatic degradation in the coal seam environment, as well as more broadly, for the first time. Although the 16S rRNA gene for Krumholzibacteriota sp. bin 3.1 (OTU_57; CSMB_1092) was able to be inferred using 16S rRNA genes from four closely related MAGs of the same phylum, downloaded from the NCBI Assembly database (46), further confirmation of the 16S rRNA gene specific to this organism could improve cross-study analysis in the coal seam environment as well as for other environments where similar taxa within the Krumholzibacteriota phylum may play a key ecological role.

In addition to aromatic degradation, the Krumholzibacteriota sp. represented by bin 3.1 may also compete in other ecological niches such as microbial biomass or necromass recycling, since it has a substantially higher number of glycoside hydrolase genes than the three Deltaproteobacteria MAGs (Table 2). Glycoside hydrolase enzymes are used for recycling complex carbohydrates and play an essential role in both ecosystem-scale and global carbon cycling (62). When compared against other biomass-recycling taxa from the coal seam environment (64), however, Krumholzibacteriota sp. bin 3.1 contains a slightly lower abundance of these and of other carbohydrate-active enzyme genes, suggesting that it is unlikely to be specializing in biomass recycling. Biomass recycling may, however, be used to supplement its extraction of carbon from coal as well as to obtain nitrogen and phosphorus (Table S2). Krumholzibacteriota sp. bin 3.1 may obtain nitrogen and phosphorus from biomass recycling and possibly from the coal itself, since this MAG contained a near-complete set of genes for the catabolism of benzamide and benzoyl phosphate to acetate via the benzoyl-CoA pathway (Fig. 3; Table S2).

Other genetic characteristics relevant to the ecological role and competitive success of Krumholzibacteriota sp. bin 3.1 include lower viral predation, higher multidrug efflux pumps, and flagella-driven motility (Table S2). Relative to the other MAGs described here, the Krumholzibacteriota sp. MAG contained far less CRISPR sequences and no *cas* genes and thus does not appear to experience the same degree of viral predation. Furthermore, the Krumholzibacteriota sp. contained approximately twice as many multidrug efflux pump genes relative to the other MAGs examined here, spanning three different transporter superfamilies, although primarily from the resistance/nodulation/cell division superfamily. These genes are associated with resistance to toxic metals such as cobalt, zinc, and cadmium as well as resistance to toxic hydrocarbons and other antimicrobial substances by removal from the cell. Finally, it is also the only MAG described here to contain most of the genes necessary for flagellar assembly and chemotaxis, required for locomotion in response to chemical cues, which would likely assist survival within the coal seam environment. Cell locomotion could also provide a competitive advantage over other potential aromatic compound degraders such as *S. aromaticivorans*, of which the type species is nonmotile (23), and no genetic potential for this was found in the MAGs examined here either (Table S2c). Overall, these findings implicate the Krumholzibacteriota sp. bin 3.1 MAG as containing the genes for a range of competitive advantages over *S. aromaticivorans* (bins 2.2 and 2.6) and *Syntrophaceae* sp. (bin 1.1) for catabolism of monoaromatic compounds in the coal seam environment. Further studies verifying the function of this microbe in the coal seam environment, such as axenic culturing to test its response to complex carbon substrates, could improve understanding. Axenic culturing could also allow observations of cell morphology and other phenotypic traits of the organism that is represented by bin 3.1, which may aid in understanding its ability to utilize different niches within the coal seam environment. The genetic characteristics provided here may serve as a guide to assist in obtaining this taxon in pure culture.

### Krumholzibacteriota taxa may liberate recalcitrant carbon in aquatic environments globally

Within the Krumholzibacteriota phylum, dearomatization and downstream catabolism of monoaromatic compounds in anoxic environments may be relatively common (Fig. 2). Of the 40 other GenBank-sourced Krumholzibacteriota MAGs used for MLSA, 13 contained a complete or near-complete set of genes for catabolism of benzoyl-CoA to 3-hydroxypimeloyl-CoA (Table S4b) (46). The environmental sources of these MAGs are listed as a deep cold seep fluid in the South China Sea (70), a hydrothermal vent in the southwest Indian Ocean (61), and freshwater lakes in Finland, Puerto Rico, and Switzerland (53). In addition to this genetic potential for anaerobic dearomatization, aerobic dearomatization has recently been suggested regarding a Krumholzibacteriota MAG that contained one subunit of the gene encoding for benzoate/toluate 1,2-dioxygenase reductase (*benC-xylZ*) and was sourced from an oil-contaminated environment in the Persian Gulf (71). In the present study, Krumholzibacteriota sp. bin 3.1 came from a subsurface coal seam (aquifer; high-similarity MAGs recovered from four of the five Flowers-Goodale coal seam metagenomes) in the USA, and the inferred 16S rRNA gene (OTU_57; CSMB_1092) has previously been identified in amplicon sequences from other subsurface coal seams in three different geological basins in eastern Australia (Table S5). In combination, these findings suggest that the role of Krumholzibacteriota taxa merits consideration when attempting to understand biodegradation of aromatic compounds in a wide range of aquatic environments and may be of relevance when attempting to understand contributions from these environments to the global carbon cycle. Again, axenic studies of putative carbon-liberating Krumholzibacteriota would be beneficial in clarifying their phylogenetic and metabolic capabilities and their ecological functions in environments such as marine sediments, deep marine cold seep fluids, hydrothermal vents, and surface freshwaters. Indeed, this clarification could result in a clearer understanding of potential rate-limiting ecological roles of Krumholzibacteriota spp. in the subsurface coal seam environment.

### *Syntrophorhabdus aromaticivorans* in the coal seam environment

Unlike the novel Krumholzibacteriota MAG, *S. aromaticivorans* has been identified within the coal seam environment on numerous previous occasions. Indeed, the CSMB reference set presently lists six distinct taxa from the *Syntrophorhabdus* genus, and one or more of these have been identified from coal-bearing basins of eastern Australia on 23 different occasions (Table S5) (8, 32). Recently, *Syntrophorhabdus* have also been detected in the Powder River Basin, USA, and implicated as a potential primary degrader of organic matter in coal based on both their increase in abundance after algal-amendment and on the published syntrophic degradative abilities for monoaromatic compounds by the type species when grown in coculture with a methanogen (23). It should be noted, however, that only this single isolate has been described from the genus and was obtained two decades ago from anaerobic sludge in terephthalic acid manufacturing wastewater (23). It was thus unclear whether other strains of *S. aromaticivorans*, such as those identified in the coal seam environment, were capable of aromatic degradation. Taken together, these data indicate for the first time that two coal seam-sourced MAGs of *S. aromaticivorans* also have the genetic potential to directly access the carbon contained within the monoaromatic molecules of coal, such as 4-hydroxybenzoate and benzoate (Fig. 1).

In addition to the genetic potential for catabolism of monoaromatic compounds, the *S. aromaticivorans* MAGs described here have noteworthy membrane transporters, viral predation indicators, and an absence of genes for biomass recycling. Both MAGs have an unusually large array of tripartite ATP-independent periplasmic transporters, which may indicate that dicarboxylates (such as fumarate) are an important source of carbon for *S. aromaticivorans* (Fig. 3). Although neither MAG had a high completeness score (Table 1), the recovered sequences did not indicate potential for biomass recycling as an alternative source of carbon, as neither strain contained substantial numbers of genes for glycoside hydrolase and other carbohydrate enzymes. Interestingly, the Australian *S. aromaticivorans* genome (bin 2.2) contains abundant CRISPR spacers, suggesting substantial pressure on this taxon from viral predation in the Bowen Basin, eastern Australia. Viral predation has been implicated as an important process in the overlying Surat Basin (14), and numerous other taxa from this environment have been previously demonstrated to harbor substantial CRISPR spacers arrayed against a host of viruses (10, 72). In contrast, the lack of CRISPR sequences detected in the North American genome (bin 2.6) indicates little to no viral predation stress on this taxon in the Powder River Basin; however, these sequences may have simply not been recovered due to the lower completeness of this bin (Table 1). Lastly, the ability of *S. aromaticivorans* to use organic electron acceptors (9,10-anthraquinone-2,6-disulfonate) when isolated from wastewater is intriguing (23), and it may be that aromatic compounds from the coal seam environment could also act as alternative electron acceptors, although experimental work with the organism in culture would be important to confirm this speculation. Regardless, certainly for Australian strains, there is clear evidence that this species has the genetic potential to catabolize aromatic compounds from the coal seam, and if these taxa are also subject to viral predation, it may be a mechanism by which this carbon is made available to a wider range of taxa post-lysis of the *S. aromaticivorans* cells.

### *Syntrophaceae* taxa may utilize aromatic carbon in coal seams

The other Deltaproteobacteria MAG represented a *Syntrophaceae* sp. from the Powder River Basin and contained genes associated with monoaromatic degradation. Data from the CSMB reference set reveal that the *Syntrophaceae* family is commonly present within coal seams in Australia, in all of the North American coal seams in the present study, and also in the Ishikari Basin, Japan (Table S5) (8). The *Syntrophaceae* family, *Smithella propionica* specifically, is well known for its use of aliphatic hydrocarbons (26, 73). Indeed, *Smithella* spp. are well-known alkane degraders from anoxic environments such as oil reservoirs, where they can grow in syntrophy with hydrogenotrophic methanogens (26). Despite their prevalence in hydrocarbon-rich environments, *Syntrophaceae* sp. bin 1.1 represents the first indication that this family may be involved in primary degradation of aromatic substrates in the coal seam environment. Given the lower MAG completeness score and the absence of other benzoyl-CoA pathway genes in *Syntrophaceae* sp. bin 1.1, further analysis could confirm accurate annotation and genetic potential, and axenic studies of this taxon would be especially useful for validation. Although incomplete, the organism represented by *Syntrophaceae* sp. bin 1.1 appears to experience considerable pressure from viral predation, as this MAG contains a high number of CRISPR spacers relative to the other Powder River Basin MAGs examined here (Fig. 3; Table 2). Viral predation may, therefore, be an important process in the Powder River Basin, as it is in other subsurface habitats.

### Ecological functions and implications for understanding the coal seam microbiome

In terms of their life strategies in the coal seam, the organisms represented by the Krumholzibacteriota, *S. aromaticivorans*, and *Syntrophaceae* MAGs likely have stress-tolerant ecological profiles, in the sense of Grime (74), in that they contain genes for a range of metabolic functions or adaptations to aid survival in this challenging environment. Each appears to possess specialized genetic tools for the degradation of a plentiful, but difficult to access, carbon source within subsurface coal seams. Interestingly, for Krumholzibacteriota sp. bin 3.1, as well as hosting these genes for monoaromatic degradation, it hosts an array of other genes associated with accessing nutrients in moribund cells or plant material (Fig. 3; Table 2). As described earlier, coal seams are oligotrophic environments, and access to other macronutrients, such as nitrogen, in coal organic matter may be important for competition in this environment. Furthermore, while it appears that the Krumholzibacteriota organism described here may have a relatively high number of genes involved in carbohydrate metabolism, it has relatively few compared to truly ruderal taxa that occur in coal seams (14).

Importantly for scientific efforts to enhance or control methane yields from coal, the four putative aromatic-degrading taxa described here may hold key rate-limiting roles in the biodegradation of coal to methane in the subsurface. If the Krumholzibacteriota sp., the two *S*. *aromaticivorans*, and the *Syntrophaceae* sp. represented by these MAGs are all indeed capable of direct access to the carbon within coal, further study of their metabolic strategies may provide important tools for altering biodegradation of coal and other complex carbon in the subsurface.

Much of the previous research into the coal seam microbiome has centered around either descriptive studies of species distributions or the effect of nutrient mixtures in an effort to enhance gas yields. While both of these approaches are valuable, they provide comparatively little information on the function of individual microbes in these communities. In contrast, this study describes the genomes of four MAGs from the coal seam environment with likely roles extracting carbon from monoaromatic compounds. Studies that seek to elucidate processes upstream of monoaromatic degradation, involving the liberation of soluble organic matter from the insoluble coal macromolecule, would further our understanding of this unusual environment.

## Data Availability

The metagenomic data sets sourced for analysis in the current study were downloaded from the NCBI Sequence Read Archive (from BioProject accessions PRJNA330673, PRJNA291107, and PRJNA678021; sequence run accessions can be found in Table S1), the JGI Data Portal (Gold project IDs Gp0406113, Gp0406114, Gp0406115, Gp0406116, and Gp0406117), and the CSIRO Data Access Portal (doi.org/10.4225/08/5b31ca6373d48). The final contig bins (MAGs), Prokka-annotated nucleotides, Kelpie-generated 16S rRNA V4 genes (fasta format file), CSMB-referenced 16S rRNA gene table, and Python scripts produced while undertaking the described methods were uploaded to the CSIRO Data Access Portal (doi.org/10.25919/bty2-hq67). An overview of these methods can be found in Fig. S1.

## SUPPLEMENTAL MATERIAL

Supplemental material mbio.01735-23-s0001.docx Figure S1, Figure S2, and Table S1.

Table S2 mbio.01735-23-s0002.xlsx MAG annotation results.

Table S3 mbio.01735-23-s0003.xlsx CheckM and Pup full results.

Table S4 mbio.01735-23-s0004.xlsx Genomes used for MLSA.

Table S5 mbio.01735-23-s0005.xlsx CSMB cooccurrence table.

ASM does not own the copyrights to Supplemental Material that may be linked to, or accessed through, an article. The authors have granted ASM a non-exclusive, world-wide license to publish the Supplemental Material files. Please contact the corresponding author directly for reuse.

## References

[1] Hardisty PE, Clark TS, Hynes RG. 2012. Life cycle greenhouse gas emissions from electricity generation: a comparative analysis of Australian energy sources. Energies 5:872–897. doi:10.3390/en5040872

[2] Markandya A, Wilkinson P. 2007. Electricity generation and health. Lancet 370:979–990. doi:10.1016/s0140-6736(07)61253-7 17876910

[3] Schandl H, Baynes T, Haque N, Barrett D, Geschke A. 2019. Final report for GISERA project G2 - whole of life greenhouse gas emissions assessment of a coal seam gas to liquefied natural gas project in the Surat Basin, Queensland, Australia. CSIRO Aust

[4] Strąpoć D, Mastalerz M, Dawson K, Macalady J, Callaghan AV, Wawrik B, Turich C, Ashby M. 2011. Biogeochemistry of microbial coal-bed methane. Annu Rev Earth Planet Sci 39:617–656. doi:10.1146/annurev-earth-040610-133343

[5] Ritter D, Vinson D, Barnhart E, Akob DM, Fields MW, Cunningham AB, Orem W, McIntosh JC. 2015. Enhanced microbial coalbed methane generation: a review of research, commercial activity, and remaining challenges. Int J Coal Geol 146:28–41. doi:10.1016/j.coal.2015.04.013

[6] Wang H, Lin H, Rosewarne CP, Li D, Gong S, Hendry P, Greenfield P, Sherwood N, Midgley DJ. 2016. Enhancing biogenic methane generation from a brown coal by combining different microbial communities. Int J Coal Geol 154–155:107–110. doi:10.1016/j.coal.2015.12.006

[7] He X, Liu X, Nie B, Song D. 2017. FTIR and Raman spectroscopy characterization of functional groups in various rank coals. Fuel 206:555–563. doi:10.1016/j.fuel.2017.05.101

[8] Vick SHW, Greenfield P, Tran-Dinh N, Tetu SG, Midgley DJ, Paulsen IT. 2018. The Coal Seam Microbiome (CSMB) reference set, a lingua franca for the microbial coal-to-methane community. Int J Coal Geol 186:41–50. doi:10.1016/j.coal.2017.12.003

[9] Wang B, Yu Z, Zhang Y, Zhang H. 2019. Microbial communities from the Huaibei Coalfield alter the physicochemical properties of coal in methanogenic bioconversion. Int J Coal Geol 202:85–94. doi:10.1016/j.coal.2018.12.004

[10] McLeish AG, Greenfield P, Midgley DJ, Paulsen IT. 2021. Draft genome sequence of Desulfovibrio sp. strain CSMB_222, isolated from coal seam formation water. Microbiol Resour Announc 10:e0056421. doi:10.1128/MRA.00564-21 34854698 PMC8638610

[11] Johnson ER, Klasson KT, Basu R, Volkwein JC, Clausen EC, Gaddy JL. 1994. Microbial conversion of high-rank coals to methane. Appl Biochem Biotechnol 45–46:329–338. doi:10.1007/BF02941809

[12] McKay LJ, Smith HJ, Barnhart EP, Schweitzer HD, Malmstrom RR, Goudeau D, Fields MW. 2022. Activity-based, genome-resolved metagenomics uncovers key populations and pathways involved in subsurface conversions of coal to methane. ISME J 16:915–926. doi:10.1038/s41396-021-01139-x 34689183 PMC8941128

[13] Schweitzer HD, Smith HJ, Barnhart EP, McKay LJ, Gerlach R, Cunningham AB, Malmstrom RR, Goudeau D, Fields MW. 2022. Subsurface hydrocarbon degradation strategies in low- and high-sulfate coal seam communities identified with activity-based metagenomics. NPJ Biofilms Microbiomes 8:7. doi:10.1038/s41522-022-00267-2 35177633 PMC8854433

[14] Vick SHW, Greenfield P, Tetu SG, Midgley DJ, Paulsen IT. 2019. Genomic and phenotypic insights point to diverse ecological strategies by facultative anaerobes obtained from subsurface coal seams. Sci Rep 9:16186. doi:10.1038/s41598-019-52846-7 31700097 PMC6838118

[15] Carmona M, Zamarro MT, Blázquez B, Durante-Rodríguez G, Juárez JF, Valderrama JA, Barragán MJL, García JL, Díaz E. 2009. Anaerobic catabolism of aromatic compounds: a genetic and genomic view. Microbiol Mol Biol Rev 73:71–133. doi:10.1128/MMBR.00021-08 19258534 PMC2650882

[16] Foght J. 2008. Anaerobic biodegradation of aromatic hydrocarbons: pathways and prospects. J Mol Microbiol Biotechnol 15:93–120. doi:10.1159/000121324 18685265

[17] Boll M, Fuchs G. 1995. Benzoyl-coenzyme A reductase (dearomatizing), a key enzyme of anaerobic aromatic metabolism. ATP dependence of the reaction, purification and some properties of the enzyme from Thauera aromatica strain K172. Eur J Biochem 234:921–933. doi:10.1111/j.1432-1033.1995.921_a.x 8575453

[18] McLeish AG, Gong S, Greenfield P, Midgley DJ, Paulsen IT. 2022. Microbial community shifts on organic rocks of different maturities reveal potential catabolisers of organic matter in coal. Microb Ecol 84:780–793. doi:10.1007/s00248-021-01857-x 34686899

[19] C Campbell B, Gong S, Greenfield P, J Midgley D, T Paulsen I, C George S. 2021. Aromatic compound-degrading taxa in an anoxic coal seam microbiome from the Surat Basin, Australia. FEMS Microbiol Ecol 97:fiab053. doi:10.1093/femsec/fiab053 33791788

[20] Kühner S, Wöhlbrand L, Fritz I, Wruck W, Hultschig C, Hufnagel P, Kube M, Reinhardt R, Rabus R. 2005. Substrate-dependent regulation of anaerobic degradation pathways for toluene and ethylbenzene in a denitrifying bacterium, strain EBN1. J Bacteriol 187:1493–1503. doi:10.1128/JB.187.4.1493-1503.2005 15687214 PMC545613

[21] Schink B, Philipp B, Müller J. 2000. Anaerobic degradation of phenolic compounds. Naturwissenschaften 87:12–23. doi:10.1007/s001140050002 10663127

[22] Smith HJ, Schweitzer HD, Barnhart EP, Orem W, Gerlach R, Fields MW. 2021. Effect of an algal amendment on the microbial conversion of coal to methane at different sulfate concentrations from the Powder River Basin, USA. Int J Coal Geol 248:103860. doi:10.1016/j.coal.2021.103860

[23] Qiu Y-L, Hanada S, Ohashi A, Harada H, Kamagata Y, Sekiguchi Y. 2008. Syntrophorhabdus aromaticivorans gen. nov., sp. nov., the first cultured anaerobe capable of degrading phenol to acetate in obligate syntrophic associations with a hydrogenotrophic methanogen. Appl Environ Microbiol 74:2051–2058. doi:10.1128/AEM.02378-07 18281436 PMC2292594

[24] Vick SHW, Gong S, Sestak S, Vergara TJ, Pinetown KL, Li Z, Greenfield P, Tetu SG, Midgley DJ, Paulsen IT. 2019. Who eats what? Unravelling microbial conversion of coal to methane. FEMS Microbiol Ecol 95:fiz093. doi:10.1093/femsec/fiz093 31216572

[25] Kuever J. 2014. The family syntrophaceae, p 281–288. In Rosenberg E, DeLong EF, Lory S, Stackebrandt E, Thompson F (ed), The prokaryotes: deltaproteobacteria and epsilonproteobacteria. Springer, Berlin, Heidelberg.

[26] Gray ND, Sherry A, Grant RJ, Rowan AK, Hubert CRJ, Callbeck CM, Aitken CM, Jones DM, Adams JJ, Larter SR, Head IM. 2011. The quantitative significance of Syntrophaceae and syntrophic partnerships in methanogenic degradation of crude oil alkanes. Environ Microbiol 13:2957–2975. doi:10.1111/j.1462-2920.2011.02570.x 21914097 PMC3258425

[27] Campbell BC, Greenfield P, Gong S, Barnhart EP, Midgley DJ, Paulsen IT, George SC. 2022. Methanogenic archaea in subsurface coal seams are biogeographically distinct: an analysis of metagenomically-derived mcrA sequences. Environ Microbiol 24:4065–4078. doi:10.1111/1462-2920.16014 35437913 PMC9790511

[28] Ravi RK, Walton K, Khosroheidari M. 2018. MiSeq: a next generation sequencing platform for genomic analysis, p 223–232. In DiStefano JK (ed), Disease gene identification: methods and protocols. Springer, New York, New York, NY. 10.1007/978-1-4939-7471-9_12 29423801

[29] Greenfield P, Duesing K, Papanicolaou A, Bauer DC. 2014. Blue: correcting sequencing errors using consensus and context. Bioinformatics 30:2723–2732. doi:10.1093/bioinformatics/btu368 24919879

[30] Prjibelski A, Antipov D, Meleshko D, Lapidus A, Korobeynikov A. 2020. Using SPAdes de novo assembler. Curr Protoc Bioinformatics 70:e102. doi:10.1002/cpbi.102 32559359

[31] Seemann T. 2014. Prokka: rapid prokaryotic genome annotation. Bioinformatics 30:2068–2069. doi:10.1093/bioinformatics/btu153 24642063

[32] von F, Granitsiotis MS, Szalay AR, Lueders T. 2020. Next-generation sequencing of functional marker genes for anaerobic degraders of petroleum hydrocarbons in contaminated environments, p 257–276. In Boll M (ed), Anaerobic utilization of hydrocarbons, oils, and lipids. Springer International Publishing, Cham.

[33] Dick GJ, Andersson AF, Baker BJ, Simmons SL, Thomas BC, Yelton AP, Banfield JF. 2009. Community-wide analysis of microbial genome sequence signatures. Genome Biol 10:R85. doi:10.1186/gb-2009-10-8-r85 19698104 PMC2745766

[34] Van Rossum G, Drake Jr FL. 1995. Python reference manual. Centrum voor Wiskunde en Informatica Amsterdam.

[35] Virtanen P, Gommers R, Oliphant TE, Haberland M, Reddy T, Cournapeau D, Burovski E, Peterson P, Weckesser W, Bright J, et al.. 2020. SciPy 1.0: fundamental algorithms for scientific computing in Python. Nat Methods 17:261–272. doi:10.1038/s41592-019-0686-2 32015543 PMC7056644

[36] Parks DH, Imelfort M, Skennerton CT, Hugenholtz P, Tyson GW. 2015. CheckM: assessing the quality of microbial genomes recovered from isolates, single cells, and metagenomes. Genome Res 25:1043–1055. doi:10.1101/gr.186072.114 25977477 PMC4484387

[37] Kanehisa M, Sato Y, Kawashima M. 2022. KEGG mapping tools for uncovering hidden features in biological data. Protein Sci 31:47–53. doi:10.1002/pro.4172 34423492 PMC8740838

[38] Kanehisa M, Sato Y, Morishima K. 2016. BlastKOALA and GhostKOALA: KEGG tools for functional characterization of genome and metagenome sequences. J Mol Biol 428:726–731. doi:10.1016/j.jmb.2015.11.006 26585406

[39] Elbourne LDH, Tetu SG, Hassan KA, Paulsen IT. 2017. TransportDB 2.0: a database for exploring membrane transporters in sequenced genomes from all domains of life. Nucleic Acids Res 45:D320–D324. doi:10.1093/nar/gkw1068 27899676 PMC5210551

[40] Zhang H, Yohe T, Huang L, Entwistle S, Wu P, Yang Z, Busk PK, Xu Y, Yin Y. 2018. dbCAN2: a meta server for automated carbohydrate-active enzyme annotation. Nucleic Acids Res 46:W95–W101. doi:10.1093/nar/gky418 29771380 PMC6031026

[41] Couvin D, Bernheim A, Toffano-Nioche C, Touchon M, Michalik J, Néron B, Rocha EPC, Vergnaud G, Gautheret D, Pourcel C. 2018. CRISPRCasFinder, an update of CRISRFinder, includes a portable version, enhanced performance and integrates search for Cas proteins. Nucleic Acids Res 46:W246–W251. doi:10.1093/nar/gky425 29790974 PMC6030898

[42] Greenfield P, Tran-Dinh N, Midgley D. 2019. Kelpie: generating full-length “amplicons” from whole-metagenome datasets. PeerJ 6:e6174. doi:10.7717/peerj.6174 30723610 PMC6359901

[43] Apprill A, McNally S, Parsons R, Weber L. 2015. Minor revision to V4 region SSU rRNA 806R gene primer greatly increases detection of SAR11 bacterioplankton. Aquat. Microb. Ecol 75:129–137. doi:10.3354/ame01753

[44] Parada AE, Needham DM, Fuhrman JA. 2016. Every base matters: assessing small subunit rRNA primers for marine microbiomes with mock communities, time series and global field samples. Environ Microbiol 18:1403–1414. doi:10.1111/1462-2920.13023 26271760

[45] Edgar RC. 2010. Search and clustering orders of magnitude faster than BLAST. Bioinformatics 26:2460–2461. doi:10.1093/bioinformatics/btq461 20709691

[46] Sayers EW, Bolton EE, Brister JR, Canese K, Chan J, Comeau DC, Connor R, Funk K, Kelly C, Kim S, Madej T, Marchler-Bauer A, Lanczycki C, Lathrop S, Lu Z, Thibaud-Nissen F, Murphy T, Phan L, Skripchenko Y, Tse T, Wang J, Williams R, Trawick BW, Pruitt KD, Sherry ST. 2022. Database resources of the national center for biotechnology information. Nucleic Acids Res 50:D20–D26. doi:10.1093/nar/gkab1112 34850941 PMC8728269

[47] Tamura K, Stecher G, Kumar S. 2021. MEGA11: molecular evolutionary genetics analysis version 11. Mol Biol Evol 38:3022–3027. doi:10.1093/molbev/msab120 33892491 PMC8233496

[48] Inkscape Project. 2022. Inkscape (1.1.2)

[49] Adobe Inc. 2021. Adobe Illustrator (26.0.2)

[50] Hug LA, Baker BJ, Anantharaman K, Brown CT, Probst AJ, Castelle CJ, Butterfield CN, Hernsdorf AW, Amano Y, Ise K, Suzuki Y, Dudek N, Relman DA, Finstad KM, Amundson R, Thomas BC, Banfield JF. 2016. A new view of the tree of life. Nat Microbiol 1:16048. doi:10.1038/nmicrobiol.2016.48 27572647

[51] Parks DH, Chuvochina M, Rinke C, Mussig AJ, Chaumeil P-A, Hugenholtz P. 2022. GTDB: an ongoing census of bacterial and archaeal diversity through a phylogenetically consistent, rank normalized and complete genome-based taxonomy. Nucleic Acids Res 50:D785–D794. doi:10.1093/nar/gkab776 34520557 PMC8728215

[52] Dalcin Martins P, de Jong A, Lenstra WK, van Helmond N, Slomp CP, Jetten MSM, Welte CU, Rasigraf O. 2021. Enrichment of novel Verrucomicrobia, Bacteroidetes, and Krumholzibacteria in an oxygen-limited methane- and iron-fed bioreactor inoculated with Bothnian Sea sediments. Microbiologyopen 10:e1175. doi:10.1002/mbo3.1175 33650794 PMC7914226

[53] Buck M, Garcia SL, Fernandez L, Martin G, Martinez-Rodriguez GA, Saarenheimo J, Zopfi J, Bertilsson S, Peura S. 2021. Comprehensive dataset of shotgun metagenomes from oxygen stratified freshwater lakes and ponds. Sci Data 8:131. doi:10.1038/s41597-021-00910-1 33990618 PMC8121793

[54] Murphy CL, Biggerstaff J, Eichhorn A, Ewing E, Shahan R, Soriano D, Stewart S, VanMol K, Walker R, Walters P, Elshahed MS, Youssef NH. 2021. Genomic characterization of three novel Desulfobacterota classes expand the metabolic and phylogenetic diversity of the phylum. Environ Microbiol 23:4326–4343. doi:10.1111/1462-2920.15614 34056821

[55] Vega MAP, Scholes RC, Brady AR, Daly RA, Narrowe AB, Bosworth LB, Wrighton KC, Sedlak DL, Sharp JO. 2022. Pharmaceutical biotransformation is influenced by photosynthesis and microbial nitrogen cycling in a benthic wetland biomat. Environ Sci Technol 56:14462–14477. doi:10.1021/acs.est.2c03566 36197061

[56] Bik EM, Costello EK, Switzer AD, Callahan BJ, Holmes SP, Wells RS, Carlin KP, Jensen ED, Venn-Watson S, Relman DA. 2016. Marine mammals harbor unique microbiotas shaped by and yet distinct from the sea. Nat Commun 7:10516. doi:10.1038/ncomms10516 26839246 PMC4742810

[57] Storey MA, Andreassend SK, Bracegirdle J, Brown A, Keyzers RA, Ackerley DF, Northcote PT, Owen JG. 2020. Metagenomic exploration of the marine sponge Mycale hentscheli uncovers multiple polyketide-producing bacterial symbionts. mBio 11:e02997-19. doi:10.1128/mBio.02997-19 32209692 PMC7157528

[58] Robbins SJ, Singleton CM, Chan CX, Messer LF, Geers AU, Ying H, Baker A, Bell SC, Morrow KM, Ragan MA, Miller DJ, Forêt S, Voolstra CR, Tyson GW, Bourne DG, ReFuGe2020 Consortium. 2019. A genomic view of the reef-building coral Porites lutea and its microbial symbionts. Nat Microbiol 4:2090–2100. doi:10.1038/s41564-019-0532-4 31548681

[59] Wang W, Tao J, Yu K, He C, Wang J, Li P, Chen H, Xu B, Shi Q, Zhang C. 2021. Vertical stratification of dissolved organic matter linked to distinct microbial communities in subtropic estuarine sediments. Front Microbiol 12:697860. doi:10.3389/fmicb.2021.697860 34354693 PMC8329499

[60] Bowers RM, Kyrpides NC, Stepanauskas R, Harmon-Smith M, Doud D, Reddy TBK, Schulz F, Jarett J, Rivers AR, Eloe-Fadrosh EA, et al.. 2017. Minimum information about a single amplified genome (MISAG) and a metagenome-assembled genome (MIMAG) of bacteria and archaea. Nat Biotechnol 35:725–731. doi:10.1038/nbt.3893 28787424 PMC6436528

[61] Zhong Y-W, Zhou P, Cheng H, Zhou Y-D, Pan J, Xu L, Li M, Tao C-H, Wu Y-H, Xu X-W. 2022. Metagenomic features characterized with microbial iron oxidoreduction and mineral interaction in southwest Indian ridge. Microbiol Spectr 10:e0061422. doi:10.1128/spectrum.00614-22 36286994 PMC9769843

[62] Berlemont R, Martiny AC. 2016. Glycoside hydrolases across environmental microbial communities. PLOS Comput Biol 12:e1005300. doi:10.1371/journal.pcbi.1005300 27992426 PMC5218504

[63] Rosewarne CP, Greenfield P, Li D, Tran-Dinh N, Midgley DJ, Hendry P. 2013. Draft genome sequence of Methanobacterium sp. maddingley, reconstructed from metagenomic sequencing of a methanogenic microbial consortium enriched from coal-seam gas formation water. Genome Announc 1:e00082-12. doi:10.1128/genomeA.00082-12 PMC3569273 23405289

[64] Vick SHW, Greenfield P, Pinetown KL, Sherwood N, Gong S, Tetu SG, Midgley DJ, Paulsen IT. 2019. Succession patterns and physical niche partitioning in microbial communities from subsurface coal seams. iScience 12:152–167. doi:10.1016/j.isci.2019.01.011 30685711 PMC6354743

[65] Rosewarne CP, Greenfield P, Li D, Tran-Dinh N, Bradbury MI, Midgley DJ, Hendry P. 2013. Draft genome sequence of Clostridium sp. maddingley, isolated from coal-seam gas formation water. Genome Announc 1:e00081-12. doi:10.1128/genomeA.00081-12 PMC3569312 23405323

[66] Schink B. 2006. Syntrophic associations in methanogenic degradation, p 1–19. In Overmann J (ed), Molecular basis of symbiosis. Springer, Berlin, Heidelberg. 10.1007/3-540-28221-1_1 16623386

[67] Jousset A, Bienhold C, Chatzinotas A, Gallien L, Gobet A, Kurm V, Küsel K, Rillig MC, Rivett DW, Salles JF, van der Heijden MGA, Youssef NH, Zhang X, Wei Z, Hol WHG. 2017. Where less may be more: how the rare biosphere pulls ecosystems strings. ISME J 11:853–862. doi:10.1038/ismej.2016.174 28072420 PMC5364357

[68] Pascoal F, Costa R, Magalhães C. 2021. The microbial rare biosphere: current concepts, methods and ecological principles. FEMS Microbiol Ecol 97:fiaa227. doi:10.1093/femsec/fiaa227 33175111

[69] Sogin ML, Morrison HG, Huber JA, Mark Welch D, Huse SM, Neal PR, Arrieta JM, Herndl GJ. 2006. Microbial diversity in the deep sea and the underexplored “rare biosphere”. Proc Natl Acad Sci U S A 103:12115–12120. doi:10.1073/pnas.0605127103 16880384 PMC1524930

[70] Zhang H, Wang M, Wang H, Chen H, Cao L, Zhong Z, Lian C, Zhou L, Li C. 2022. Metagenome sequencing and 768 microbial genomes from cold seep in South China Sea. Sci Data 9:480. doi:10.1038/s41597-022-01586-x 35933411 PMC9357000

[71] Rezaei Somee M, Dastgheib SMM, Shavandi M, Ghanbari Maman L, Kavousi K, Amoozegar MA, Mehrshad M. 2021. Distinct microbial community along the chronic oil pollution continuum of the Persian Gulf converge with oil spill accidents. Sci Rep 11:11316. doi:10.1038/s41598-021-90735-0 34059729 PMC8166890

[72] Vick SHW, Greenfield P, Willows RD, Tetu SG, Midgley DJ, Paulsen IT. 2020. Subsurface Stappia: success through defence, specialisation and putative pressure-dependent carbon fixation. Microb Ecol 80:34–46. doi:10.1007/s00248-019-01471-y 31828390

[73] Zengler K, Richnow HH, Rosselló-Mora R, Michaelis W, Widdel F. 1999. Methane formation from long-chain alkanes by anaerobic microorganisms. Nature 401:266–269. doi:10.1038/45777 10499582

[74] Grime JP. 1977. Evidence for the existence of three primary strategies in plants and its relevance to ecological evolutionary theory. Am Nat 111:1169–1194. doi:10.1086/283244

