# Supplementary material for: Krumholzibacteriota and Deltaproteobacteria contain rare genetic potential to liberate carbon from monoaromatic compounds in subsurface coal seams
Source: mBio. 2024 Feb 12;15(3):e01735-23. doi: 10.1128/mbio.01735-23 (PMC10936416; doi:10.1128/mbio.01735-23)
Supplement: Supplemental material — Figure S1, Figure S2, and Table S1. [file mbio.01735-23-s0001.docx]

**Supplementary Data**

*Table of contents*

**Figure S1:** Metagenome-assembled genome method overview, including taxonomic classification steps.

**Figure S2:** Maximum likelihood phylogeny of bin 3.1 within the Fibrobacteres-Chlorobiota-Bacteroidota (FCB) superphylum using multilocus sequence analysis (MLSA).

**Table S1:** Coal seam formation water metagenomes used in this study, as well as in Campbell et al 2022.

**References**

***Please note:***

Tables S2-S5 can be downloaded as separate files from the Supplementary Data section of the online publication.

Contig bins 1.1, 2.2, 2.6, and 3.1, Prokka annotations of these, Python scripts used in the methods, as well as 16S rRNA gene OTU sequences and a data table including corresponding CSMB reference set numbers, can all be found on the CSIRO Data Access Portal: [doi.org/10.25919/d4nf-8f32](https://doi.org/10.25919/d4nf-8f32).


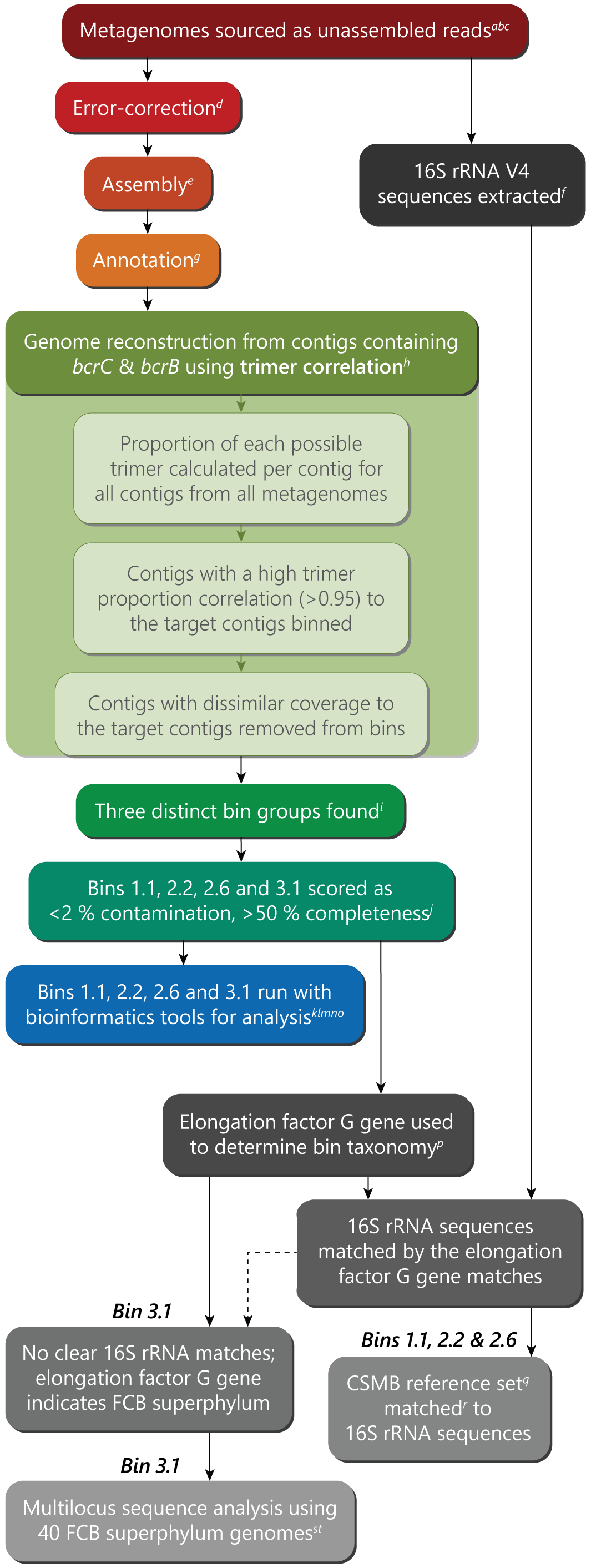


**Figure S1:** Metagenome-assembled genome method overview, including taxonomic steps. ‘16S rRNA’ = the V4 region of the 16S ribosomal RNA gene.

*^a^*Sequence Read Archive ([ncbi.nlm.nih.gov/sra](https://www.ncbi.nlm.nih.gov/sra)); *^b^*Joint Genome Institute ([img.jgi.doe.gov/](https://img.jgi.doe.gov/)); *^c^*Commonwealth Scientific and Industrial Research Organisation Data Access Portal ([data.csiro.au/](https://data.csiro.au/)); *^d^*Blue [1]; *^e^*metaSPAdes [2]; *^f^*Kelpie [3]; *^g^*Prokka [4]; *^h^*[5]; *^i^*Pup ([github.com/PaulGreenfieldOz/WorkingDogs](https://github.com/PaulGreenfieldOz/WorkingDogs)); *^j^*CheckM [6]; *^k^*BlastKOALA [7]; *^l^*TransportDB [8]; *^m^*CRISPRCasFinder [9]; *^n^*dbCAN [10]; *^o^*ISSaga [11]; *^p^*NCBI BLASTn ([blast.ncbi.nlm.nih.gov/Blast.cgi#](https://blast.ncbi.nlm.nih.gov/Blast.cgi)); *^q^* [12]; *^r^*USEARCH [13]; *^s^*MEGA11 [14]; *^t^*FigTree ([tree.bio.ed.ac.uk/software/figtree/](http://tree.bio.ed.ac.uk/software/figtree/)).

**
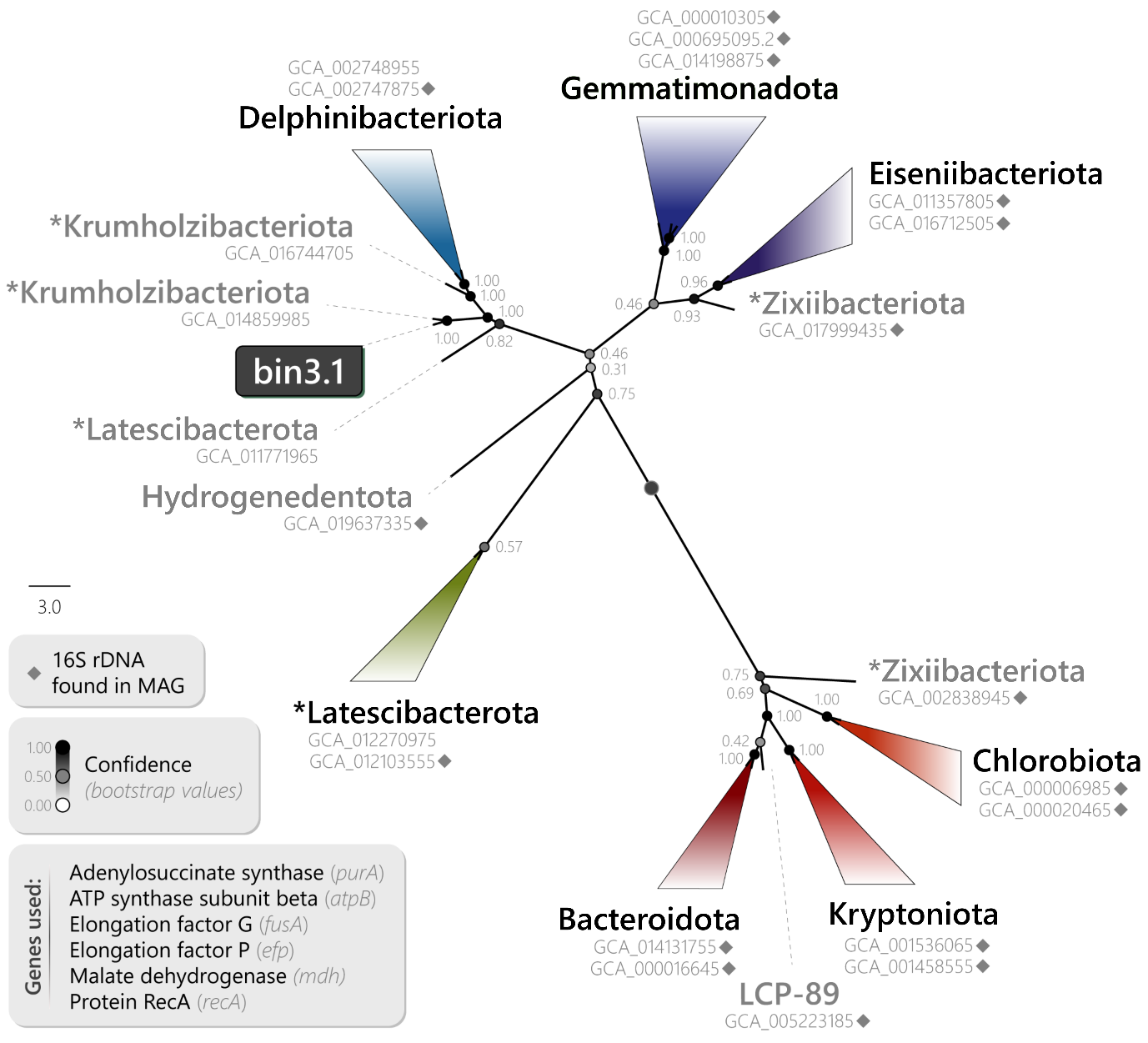
Figure S2:** Maximum likelihood phylogeny of bin 3.1 within the Fibrobacterota-Chlorobiota-Bacteroidota (FCB) superphylum using multilocus sequence analysis (MLSA).

Although a total of 37 representative FCB superphylum genomes were used in the initial analysis, only 22 of these are shown above because not all genomes contained all of the selected housekeeping genes. Multiple representative genomes from the Aegiribacteria, Cloacimonadota and Fermentibacterota phyla were included in the analysis but none appear on this tree as each were missing one or more genes from the final selection of concatenated housekeeping genes (Supplementary Table S4). The Eiseniibacteriota genome assembly GCA_011357805 is noted in the NCBI Assembly database [15] as being suppressed.

| **Table S1:** Coal seam formation water metagenomes used in this study, as well as in Campbell et al 2022 [16]. | | | | |
| --- | --- | --- | --- | --- |
| **Dataset name used in this study** | **NCBI sequence run¹** | **Geological basin, country** | **Formation** | **Submitter & year** |
|  | **IMG Genome ID²** |  |  |  |
| CentralAppalachian_89 | SRR3952189¹ | Appalachian Basin, USA | Pocahontas No. 3 Coal Seam | D. Ross, 2016 |
| Surat_01 | Not applicable³ | Surat Basin, Australia | Walloon Subgroup | Greenfield et al. 2019 & this study |
| Surat_02 | Not applicable³ |  |  |  |
| Surat_06 | SRR2132206¹ |  |  | D. Parks, 2015 |
| Bowen_03 | Not applicable³ | Bowen Basin, Australia | Bandanna Formation | Greenfield et al. 2019 & this study |
| PowderRiver_10 | SRR13051710¹ | Powder River Basin, USA | Nance coal seam | M.W. Fields, 2020 |
| PowderRiver_09 | SRR13051709¹ |  | Flowers-Goodale coal seam |  |
| PowderRiver_50ᵃ | SRR12666050¹ |  |  | E. Barnhart, 2019 |
|  | 3300037286² |  |  |  |
| PowderRiver_40ᵇ | SRR11844740¹ |  |  |  |
|  | 3300037047² |  |  |  |
| PowderRiver_37ᶜ | SRR11844737¹ |  |  |  |
|  | 3300037046² |  |  |  |
| PowderRiver_85ᶜ | SRR11844785¹ |  |  |  |
|  | 3300037048² |  |  |  |
| PowderRiver_84 | SRR11844784¹ |  | Terret coal seam |  |
|  | 3300036987² |  |  |  |
| PowderRiver_08 | SRR13051708¹ |  |  | M.W. Fields, 2020 |
| ¹Available from the NCBI SRA database ([15]; [ncbi.nlm.nih.gov/sra](https://www.ncbi.nlm.nih.gov/sra)) | | | | |
| ²Available from JGI Genome Portal ([17]; [genome.jgi.doe.gov/portal/](https://genome.jgi.doe.gov/portal/)) | | | | |
| ³Available from the CSIRO Data Access Portal ([data.csiro.au/collection/csiro:33617v1](https://data.csiro.au/collection/csiro:33617v1)): ‘W1’ = Surat_01; ‘W2’ = Bowen_03; ‘W3’ = Surat_02 [3] | | | | |
| ᵃFrom well FGM in the Flowers-Goodale Coal Seam | | | | |
| ᵇFrom well FGP in the Flowers-Goodale Coal Seam | | | | |
| ᶜFrom well FG11 in the Flowers-Goodale Coal Seam | | | | |

**Disclaimer**

Any use of trade, firm, or product names is for descriptive purposes only and does not imply endorsement by the U.S. Government.

**References**

1. Greenfield P, Duesing K, Papanicolaou A, Bauer DC. Blue: correcting sequencing errors using consensus and context. *Bioinformatics* 2014; **30**: 2723–2732.

2. Nurk S, Meleshko D, Korobeynikov A, Pevzner PA. metaSPAdes: a new versatile metagenomic assembler. *Genome Res* 2017; **27**: 824–834.

3. Greenfield P, Tran-Dinh N, Midgley D. Kelpie: generating full-length ‘amplicons’ from whole-metagenome datasets. *PeerJ* 2019; **6**.

4. Seemann T. Prokka: rapid prokaryotic genome annotation. *Bioinformatics* 2014; **30**: 2068–2069.

5. Dick GJ, Andersson AF, Baker BJ, Simmons SL, Thomas BC, Yelton AP, et al. Community-wide analysis of microbial genome sequence signatures. *Genome Biol* 2009; **10**: R85.

6. Parks DH, Imelfort M, Skennerton CT, Hugenholtz P, Tyson GW. CheckM: assessing the quality of microbial genomes recovered from isolates, single cells, and metagenomes. *Genome Res* 2015; **25**: 1043–1055.

7. Kanehisa M, Sato Y, Morishima K. BlastKOALA and GhostKOALA: KEGG Tools for Functional Characterization of Genome and Metagenome Sequences. *J Mol Biol* 2016; **428**: 726–731.

8. Elbourne LDH, Tetu SG, Hassan KA, Paulsen IT. TransportDB 2.0: a database for exploring membrane transporters in sequenced genomes from all domains of life. *Nucleic Acids Res* 2017; **45**: D320–D324.

9. Couvin D, Bernheim A, Toffano-Nioche C, Touchon M, Michalik J, Néron B, et al. CRISPRCasFinder, an update of CRISRFinder, includes a portable version, enhanced performance and integrates search for Cas proteins. *Nucleic Acids Res* 2018; **46**: W246–W251.

10. Zhang H, Yohe T, Huang L, Entwistle S, Wu P, Yang Z, et al. dbCAN2: a meta server for automated carbohydrate-active enzyme annotation. *Nucleic Acids Res* 2018; **46**: W95–W101.

11. Varani AM, Siguier P, Gourbeyre E, Charneau V, Chandler M. ISsaga is an ensemble of web-based methods for high throughput identification and semi-automatic annotation of insertion sequences in prokaryotic genomes. *Genome Biol* 2011; **12**: R30.

12. Vick SHW, Greenfield P, Tran-Dinh N, Tetu SG, Midgley DJ, Paulsen IT. The Coal Seam Microbiome (CSMB) reference set, a *lingua franca* for the microbial coal-to-methane community. *Int J Coal Geol* 2018; **186**: 41–50.

13. Edgar RC. Search and clustering orders of magnitude faster than BLAST. *Bioinformatics* 2010; **26**: 2460–2461.

14. Tamura K, Stecher G, Kumar S. MEGA11: Molecular Evolutionary Genetics Analysis Version 11. *Mol Biol Evol* 2021; **38**:3022–3027.

15. Sayers EW, Bolton EE, Brister JR, Canese K, Chan J, Comeau DC, et al. Database resources of the national center for biotechnology information. *Nucleic Acids Res* 2022; **50**: D20–D26.

16. Campbell BC, Greenfield P, Gong S, Barnhart EP, Midgley DJ, Paulsen IT, et al. Methanogenic archaea in subsurface coal seams are biogeographically distinct: an analysis of metagenomically-derived mcrA sequences. *Environ Microbiol* 2022; **24**: 4065–4078.

17. Grigoriev IV, Nordberg H, Shabalov I, Aerts A, Cantor M, Goodstein D, et al. The Genome Portal of the Department of Energy Joint Genome Institute. *Nucleic Acids Res* 2012; **40**: D26–D32.
